# Identifying Childhood Risk Factors for Hepatitis B with a Focus on Vertical Transmission

**DOI:** 10.3390/diseases12090215

**Published:** 2024-09-16

**Authors:** Lorenza Forna, Ancuta Lupu, Laura Bozomitu, Gabriela Paduraru, Camelia Cojocariu, Carmen Anton, Irina Girleanu, Cristina Maria Muzica, Anca Trifan

**Affiliations:** 1 Pediatrics—“Sf. Maria” Clinical Emergency Children’s Hospital, Iassy 700309, Romania; lorenza.donea@yahoo.ro (L.F.); anca_ign@yahoo.com (A.L.); gaby_spulber@yahoo.com (G.P.); 2 Faculty of General Medicine, University of Medicine and Pharmacy “Gr. T. Popa”, Iași 700115, Romania; camelia.cojocariu@umfiasi.ro (C.C.); carmen.anton@umfiasi.ro (C.A.); irina.girleanu@umfiasi.ro (I.G.); lungu.christina@yahoo.com (C.M.M.); ancatrifan@yahoo.com (A.T.); 3 Department of Clinical Gastroenterology, “Sf. Spiridon” Clinical Emergency Hospital, Iași 700111, Romania

**Keywords:** HBV infection, childhood risk factors, vertical transmission, vaccination

## Abstract

Background: Despite worldwide vaccination efforts, Hepatitis B virus (HBV) infection remains a significant global health burden, particularly in regions where vertical transmission is prevalent. Given Romania’s history as an endemic area for hepatitis B from the 1990s until the early 2000s and the previously high infection rates among children, it is crucial to continually evaluate HBV infection in this population to monitor current trends, assess the long-term impact of vaccination programs, and address any remaining gaps in prevention and treatment efforts. This study aims to identify childhood risk factors associated with HBV acquisition, examining the role of maternal HBV status in child HBV infection, focusing on vertical transmission among a cohort of 654 children, with maternal infection as the independent variable and child infection as the dependent variable. Methods: We assessed potential risk factors and vaccination coverage among these children. The cohort included 148 children who tested positive for chronic hepatitis B from those 654 tested for HBsAg. Anamnestic data and vaccination history were analyzed, with particular attention to birth type and surgical interventions. Results: Of the 148 HBV-positive children, 80.4% were delivered naturally. Among these, 130 had received hepatitis B vaccination, and 5 were also given hepatitis B immunoglobulin at birth, 4 of whom were born via cesarean section. In the control group, comprising 418 vaccinated children, a lesser proportion were unvaccinated (2.2%). Documented surgical interventions included general and dental surgeries, as well as a single blood transfusion. Conclusions: The study emphasizes the need for comprehensive vaccination strategies and illuminates potential correlations between birth type and vaccination status with childhood HBV infection. Crucially, it highlights the necessity of diligent monitoring and treatment of pregnant women with HBV to prevent vertical transmission as effectively as possible.

## 1. Introduction

Hepatitis B virus (HBV) infection remains a significant global health issue and is the precursor stage of cirrhosis and the 10th leading cause of mortality globally. It should be noted that the prevalence and incidence of chronic HBV infection are higher in underdeveloped countries than in more economically developed ones, specifically 7.4 and 9.2 times higher, respectively [1]. However, this is inversely proportional to the diagnosis rates of this pathology: in developed countries, the diagnosis rate is 18%, compared to other regions where it drops to 0.8% [2]. Population migration has led to an increase in prevalence in certain areas, for example, from Southeast Asia to the United States. Nevertheless, the US still maintains a low prevalence, below 2%. Africa is the main region with an HBsAg (Hepatitis B surface antigen) prevalence of over 3% in children under 5 years old [1].

Currently, there are 189 countries that include hepatitis B vaccination in their national immunization programs. According to WHO, globally, only 43% of newborns receive the first dose of the vaccine at birth, while in the African region, coverage is 6% [3].

Romania experienced a notably high incidence of hepatitis B, particularly affecting children. From 1990 to 2002, the country recorded an annual hepatitis B virus (HBV) infection rate of 10–50 cases per 100,000 population [4]. Comparative studies in 1990 placed Romania as an endemic area for hepatitis B within Europe [5,6]. A specific study conducted in Bucharest between April and July 1990 revealed high HBV prevalence across all age groups. About 47% of adults and 40% of children aged 0–16 years tested positive for at least one HBV marker (HBsAg and/or anti-HBc—antibody to the hepatitis B core antigen). The prevalence was even higher among infants under 3 years in orphanages, with 55% testing positive for these markers. Additionally, nearly 8% of pregnant women were found to be HBsAg-positive, indicating widespread exposure and infection rates within these populations [5]. Pitigoi et al. examined the change in HBV infection rates in Romania from the late 1980s to 2005, highlighting the effectiveness of vaccination. As a result, the overall incidence of hepatitis B dramatically decreased from 43 per 100,000 in 1989 to 8.5 per 100,000 in 2004, with the most significant decline observed in children under 15, where incidence dropped from 81 to 11 per 100,000 population per year [7]. Administering the vaccine in maternity wards together with human hepatitis B immunoglobulin (HBIG) reduces the risk of infection to between 0.7 and 1.1%. Additionally, the efficacy of these measures demonstrates that most infections occur in the last trimester or during birth, with less than 15% occurring intrauterinely [8]. Of major interest currently are the numerous cases where, despite immunoprophylaxis at birth and delivery via cesarean section, the viral infection persists.

In the pediatric population, transmission of HBV is predominantly vertical and intrafamilial [9]. Based on the geographical area, it is accepted that those with a high prevalence (>8%) have predominantly perinatal transmission, areas with intermediate prevalence (2–8%) have horizontal, intrafamilial transmission, and finally, those with low prevalence (<2%) present transmission through sexual contact and invasive procedures usually contracted during adolescence (intravenous drugs) [10].

Regarding the notion of intrauterine transmission of HBV, the data are not fully elucidated, but it is noteworthy that the presence of HBsAg or viremia in infants can be transient through transplacental transfer from the mother, so 88.1% of those HBsAg+ and 64.3% of those HBV-DNA+ can eliminate it within 8–12 months. Similarly, for HBeAg (Hepatitis B e antigen), anti-HBe antibodies are up to 1 year, and for anti-HBc, antibodies are up to 2 years of age. Thus, intrauterine HBV infection can be defined as the persistence of HBsAg or viremia after 9 months despite immunoprophylaxis from birth [11]. It is considered that a high viral DNA and the presence of HBeAg during pregnancy in HBV-positive mothers would represent a risk factor for transmitting the infection to the child from the intrauterine period. In fact, this, and the presence of genotype C of the virus in mothers, would explain the inefficiency of complete immunization of the newborn [12].

Additionally, regarding the breastfeeding of infants by HBsAg-positive mothers, it has been demonstrated that breast milk is safe and does not contribute to the transmission of the virus [13]. However, careful monitoring is required for mothers with a viral load greater than 10^6 copies/mL in serum or in cases of breaches in continuity either at the nipple or at the oral mucosa of the newborn.

In some cases, even with the administration of antiviral treatment focused solely on seroconversion in the HBe system, the infection persists. This indicates that although HBeAg is correlated with a high viral load, revealing active viral replication, the essential factor is the viremia of the pregnant woman [11].

Until the 2000s, Romania was part of an endemic area for HBV infection in children. The vaccination, introduced into the mandatory immunization schedule in 1995, contributed significantly to the reduction in cases in the pediatric population. However, generational differences still exist, as mothers born before the 1990s with HBV infection are still at risk of transmitting the virus to their children, both vertically and horizontally. Therefore, this retrospective study aims to highlight the risk factors for HBV infection in children, with a particular focus on vertical transmission. The main objectives of this study include determining and analyzing the specific risk factors in children that contribute to the contraction and spread of Hepatitis B, as well as those that may influence the disease’s progression. A particular focus is on vertical transmission, which is recognized as the most common mode of transmission in the pediatric population.

## 2. Materials and Methods

In this retrospective analysis, data were gathered from a tertiary healthcare facility located in northeastern Romania. Among 654 children tested for HBsAg, 148 were diagnosed with chronic HBV infection. This study utilized a longitudinal discriminant analysis to monitor disease progression and forecast outcomes. The findings presented here are part of interim results from a PhD research project exploring the risk factors, including vertical transmission of HBV in children treated at the ‘Sf. Maria’ Hospital in Iasi. The data were collected for the period between 2011 and 2019, with each patient having at least one medical visit recorded during this interval. The patients come from various regions of the country but predominantly from the eastern part of Romania, representing both urban and rural areas. Chronic HBV infection was defined by the continued presence of HBsAg for a period of at least six months. HBsAg testing in these children was likely prompted by maternal HBV status, clinical symptoms, or other hospitalization reasons, during which chronic HBV infection was diagnosed in the Pediatric Gastroenterology section. This retrospective descriptive study analyzed medical records from children in northeastern Romania, using both descriptive statistics and logistic regression to elucidate the relationship between maternal HBV status or other risk factors and child HBV infection.

Inclusion Criteria:

- Children aged 0 to 18 years at their initial pediatric consultation for chronic HBV infection.
- Evidence of HBsAg for a minimum duration of six months.

Exclusion Criteria:

- Concurrent infections with HIV (Human Immunodeficiency Virus), HCV (Hepatitis C Virus), or HDV (Hepatitis D Virus), or absence of initial data, were grounds for exclusion.

The descriptive characterization of the studied cohort was based on several criteria, including the mother’s viral status, place of birth, type of delivery, completeness of the vaccination schedule, and administration of hepatitis B immunoglobulin. Risk factors were derived from the patient’s family and personal medical history, such as frequent hospitalizations, blood transfusions, surgeries, dental procedures, trauma, shared personal items, immunosuppression status, and absent or incomplete hepatitis B vaccination at birth.

Statistical Analysis: Statistical assessments were performed using SPSS version 29.0. Qualitative data were analyzed through frequency distributions, while quantitative data were evaluated using descriptive statistics, including means and standard deviations. A threshold for statistical significance was established at *p* < 0.05. The Pearson Chi-square test is used to evaluate whether there is a significant association between two categorical variables. We employed this analysis to test the independence of variables within contingency tables. Additionally, we used logistic regression analysis for risk factors. The Odds Ratio (OR) is a measure of the association between an exposure and an outcome; a value greater than 1 indicates a higher likelihood of the outcome occurring with the exposure.

## 3. Results

### 3.1. Prevalence by Age Groups

This study examines a cohort of 654 children who were tested for HBV, with 148 testing positive for chronic viral hepatitis B. These children range (Table 1) in age from 4 months to 18 years. Within the positive group, there is a slight predominance in the 2–5-year age bracket, while the majority (38.5%) of the entire cohort were tested between the ages of 0 and 1 year. Pearson Chi-square = 30.239, *p* < 0.001.

This table provides a structured breakdown of the data, indicating a statistically significant difference in the distribution between active and control groups across different age ranges.

### 3.2. Social Status

The distribution of patients in the active group based on social status (Table 2) reveals a significant number of children in foster care (17), as well as two adopted. This highlights issues such as the abandonment of newborns in maternity wards for various reasons or reasons such as the revocation of parental rights, a problem associated with low living standards and the inability to care for children, vices within the family, domestic violence, or the death of both parents.

### 3.3. Type of Birth

Regarding the type of birth (Table 3), there is a clear majority of natural births (80.4% in the active group and 64.8% in the control group), which may be beneficial for both the postpartum progress of the mother and the newborn, of course, as a general process, except for the risk of vertical transmission of HBV.

### 3.4. Family History

After analyzing the data from the study, 121 children with chronic HBV infection come from HBsAg-positive mothers, while 27 are from HBsAg-negative mothers (Table 4). In the control group, 14 children have HBV + HDV coinfection from HBV-positive mothers, 407 are negative for HBsAg, and 85 have no data regarding this aspect or have other pathologies. The fact that 81.8% of the positive children have vertical transmission of hepatitis B virus suggests incomplete immunization or improper prenatal care of mothers during pregnancy, before pregnancy, and after birth. Among these, 26 mothers were diagnosed after pregnancy, many years after birth, even after the child’s diagnosis, and one of the mothers was positive during pregnancy. It is notable that 10 patients have grandparents with chronic viral hepatitis B on the maternal side, indicating the presence of three generations with this pathology, transmitted vertically. Additionally, 62 children have siblings with HBV infection, and 5 have both healthy and sick siblings. Practically, these siblings (the largest group being of five siblings) are 41.9% positive for HBV, pointing to the increased virulence of the mother during pregnancy and at the time of birth. From the anamnestic data, no mother followed antiviral treatment in the last weeks of pregnancy.

Regarding the family history on the father’s side, it was observed that a significant number of 21 fathers in the active group have HBV infection, with 4 of them being the only sick parents, while the remaining 17 are positive along with mothers. This could be interpreted as intrafamilial transmission for these four children without definitive tests proving the health status of the mothers. The control group predominantly consists of families with a single child; of those with siblings, 45.8% are healthy, and only 16 have siblings with HBV, with 14 of these having HBV+HDV infection.

### 3.5. Vaccination

**Figure 1:**
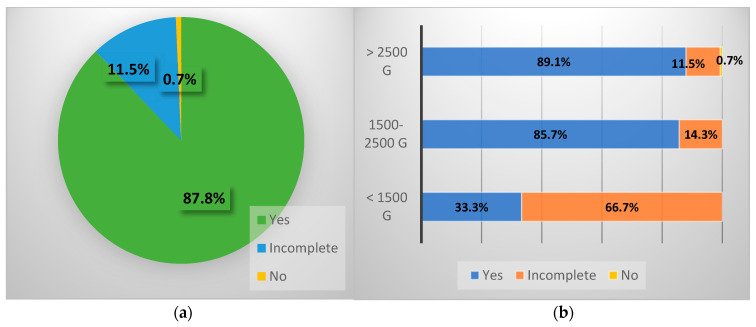
(**a**) Prevalence of vaccination in active lot. (**b**) Prevalence of vaccination in active lot based on weight status at birth.

In the active group, according to anamnestic data, 130 children were vaccinated for hepatitis B virus (Table 5), 17 were incompletely vaccinated, and one was not vaccinated at all. In the control group, 418 children were vaccinated, while 11 children were not vaccinated at all. In the positive group, it is noteworthy that out of the 130 vaccinated (Figure 1), 5 also received hepatitis B immunoglobulin at birth, and 4 of them were born via cesarean section.

### 3.6. HBIG Administration

In our cohort, only 5 patients received HBIG in the first hours after birth (3.4%), 52 did not receive it (35.1%), while for the remaining 61.5%, the caregivers stated that they did not know about this immunization or whether the newborn received it (Table 6). These results indicate a significant association between the lack of HBV immunoglobulin administration at birth and the incidence of HBV infection in children.

### 3.7. Breastfeeding

Regarding breastfeeding in HBV infection, there are few studies, but most have reached a consensus at present, specifically on the possibility of breastfeeding the newborn/infant by the infected mother.

**Figure 2:**
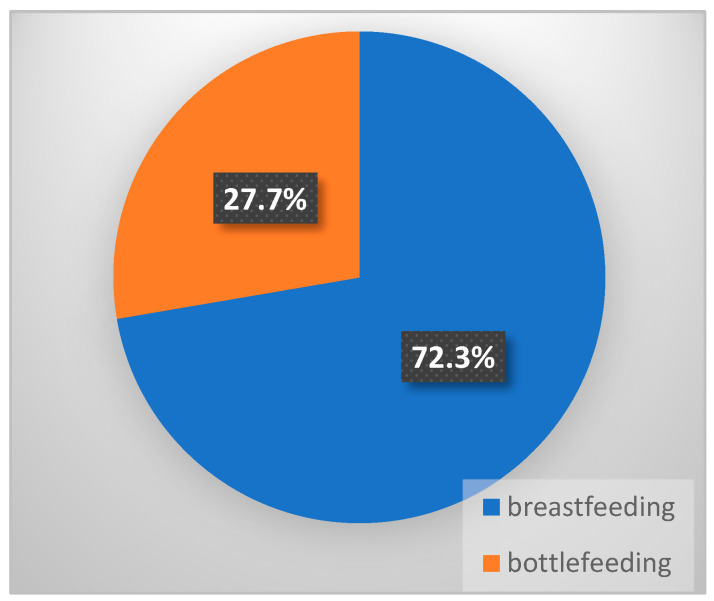
Prevalence of breastfeeding in HBV infected children.

In our active group, 107 (72.3%) children were breastfed, and 41 (27.7%) received formula (Figure 2), which is encouraging from nutritional, immunological, and psycho-emotional perspectives.

### 3.8. Smoking Environment

The data regarding the impact of a smoking environment on HBV infection in children are presented in Table 7. A total of 37.3% of the total cohort was exposed to cigarette smoke, while 54.1% did not smoke or have smokers in the family. Of the 506 patients in the control group, only 479 had data regarding smoking.

### 3.9. Risk Factors—Surgical Interventions

From the analysis of the active group, it can be observed (Table 8) that most risk factors, aside from vertical transmission, are represented by surgical interventions, including 15 general surgeries, five dental surgeries, and one blood transfusion. It is well known that until recently, the main mode of transmission for the hepatitis B virus was through infected blood via improperly sterilized instruments (injections, manicures, cosmetic procedures, tattoos, IV drugs, piercings). However, today, there are single-use needles, screening of blood to be transfused, and accessible protective equipment.

Univariate analysis (Odds Ratio) and multivariate analysis through binary logistic regression regarding the risk factors showed the following results (Table 9): there is a highly significant association between having an HBsAg+ mother and the risk of HBV infection in children. Children of HBsAg+ mothers are 152.7 times more likely to be infected compared to those with HBsAg- mothers. Regarding immunization, there is no significant association between incomplete or no vaccination and the risk of HBV infection in children based on this data. These results are certainly influenced by the predominance of vertical transmission and the fact that immunization was not completed at birth.

There is a significant association between natural birth and the risk of HBV infection in children. Children born naturally are 1.7 times more likely to be infected compared to those born via cesarean section. In our study, we found a statistically significant correlation between hepatitis B virus infection and the incidence of respiratory, endocrine, nutritional, metabolic, or renal diseases (*p* < 0.001 **). This association may contribute to a negative progression of HBV infection due to compromised immune function in these patients. Additionally, our data indicate that children who have undergone surgical interventions are 2.1 times more likely to contract HBV infection, highlighting the potential risks associated with surgical procedures in this population.

## 4. Discussion

In our study, the finding that 81.8% of children with hepatitis B acquired the infection through vertical transmission highlights potential problems with either incomplete immunization or inadequate prenatal care before, during, and after pregnancy. Most children are family members (87.2%), but the 11.5% from foster care and 1.4% adopted to highlight the fact that many newborns are abandoned in maternity wards, have no documented history, were not monitored during pregnancy, or are more likely to come from sick parents, with addictions or who are minors themselves, originating from disadvantaged environments.

### 4.1. Type of Birth

Natural birth predominates in this study (80.4%). However, in the case of HBV infection, the situation is disadvantageous for the child, as the risk of vertical transmission appears to be much higher (28%) in natural births, according to Wong et al., compared to cesarean deliveries, which have a risk of 10.8% [14]. Nevertheless, the data are insufficient; some studies indicate that there are no significant differences between the two types of birth or that surgical intervention is not advisable in the context of a high viral load, precisely to avoid increasing the risk of transmission through infected blood [15]. Additionally, various minimally invasive procedures such as chorionic villus sampling, amniocentesis, or prolonged labor should be avoided during pregnancy.

### 4.2. Vaccination and HBIG

The global coverage of hepatitis B immunization is 39%, according to 2015 data [2]. The highest vaccination rate is in the American continent, while the lowest rate is in the region with the highest prevalence, Africa (10%). In Romania, vaccination has been mandatory since 1995, but there are no precise data regarding the vaccination rate for newborns.

It is already known that the most effective way to prevent the vertical transmission of hepatitis B virus infection is by administering the vaccine and hepatitis B immunoglobulin (HBIG) at birth, reducing the risk from 5–25% to 0.7–1.1% [11]. However, the risk is particularly present if the mother was not monitored during pregnancy and did not receive the necessary treatment. The HBV vaccination schedule includes a monovalent dose within the first 24 h after birth, followed by combined vaccine doses at 2 months, 4 months, and 11 months for all children [8,11]. For newborns from HBsAg mothers, administering a dose of hepatitis B immunoglobulin (HBIG) at birth further, alongside the vaccine, reduces the transmission risk to 6% for HBeAg-positive women and to 1% for HBeAg-negative women [16]. In our study, children who received HBIG were administered the immunoglobulin within the first 12 h of birth. Mothers who were informed that their newborn would need this immunization purchased the product from pharmacies before giving birth. This occurs because, theoretically, all mothers are tested for the HBs antigen during pregnancy. However, there are many cases where women in labor present to the emergency department at the time of delivery without having been monitored during pregnancy, especially those from rural areas or those who claim to have been registered but are unaware of their viral status. In our country, until 2019, HBIG was administered to the newborn of an HBsAg-positive mother only if the family purchased the product from the pharmacy, as it was not supplied by the maternity hospitals. The cost was considerable, frequently making it challenging for families with modest incomes to access immunoglobulin. However, for the five patients with failed immunoprophylaxis, we can consider the causes of increased infectiousness before birth; none of the mothers who knew they were infected were informed (declaratively) about possible antiviral treatment during pregnancy, in addition to active and passive immunization. The low percentage of HBIG administration indicated misinformation among pregnant women, a deficient interdisciplinary relationship between family doctors, obstetricians, neonatologists, and gastroenterologists, as well as the lack of a national program to provide immunoglobulin in maternity hospitals. Fortunately, changes regarding passive immunization at birth (HBIG) to the therapeutic protocol for chronic viral hepatitis B were made in 2019 and implemented after 2020. Currently, hepatitis B immunoglobulin is available at birth in maternity hospitals, eliminating the need for parents to procure it themselves.

HBIG is a solution made from human plasma that contains anti-HB antibodies at high titers. It can be administered along with the hepatitis B vaccine but in different sites and should not be mixed with other solutions [17]. There are studies that have proven the efficacy of HBIG in pregnant women infected with HBV, showing a decrease in viremia and the HBsAg titer within 3–7 days after administration; however, by 4 weeks, the values increased back to levels as high as before [17,18]. Thus, passive immunization of the fetus occurs through the transfer of anti-HB antibodies across the placenta. There are also studies where no decrease in maternal viremia or increase in the anti-HBs antibody titer in newborns was observed [19], which is why there is no consensus on the use of HBIG in pregnant women and its effectiveness in stopping the vertical transmission of the hepatitis B virus [20].

### 4.3. Breastfeeding

In our study, 72.3% of children were breastfed. However, it is important to monitor the maternal viral load, as an HBV DNA level ≥ 10^6 copies/mL can increase the risk of viral transmission. Mothers who are positive for HBV and did not undergo antiviral treatment during pregnancy are at a high risk of passing the virus to their infants unless preventive actions are implemented. However, breastfeeding does not significantly contribute to the transmission of HBV, provided that the infant is properly vaccinated. Unfortunately, most mothers did not know that they were already infected with HBV at the time of birth. It has also been demonstrated that colostrum has the highest level of viremia, directly proportional to the mother’s blood viremia [21]. This does not contraindicate breastfeeding in the first days if the newborn has been correctly immunized (vaccine + HBIG). Zhou et al. found in their analysis of a cohort of 546 correctly immunized children born to HBsAg-positive mothers that those who were breastfed had higher levels of anti-HBs antibodies compared to those fed with formula, who had lower levels [12,22]. Antiviral treatment is indicated in the third trimester of pregnancy if the mother’s viremia is ≥ 200,000 IU/mL and can be continued postpartum, during which breastfeeding is allowed, depending on the type of antiviral. Tenofovir has been shown to be the most effective and safe in this regard, with only 0.03% of the administered dose being transmitted through breast milk [23]. It is crucial to closely monitor both the mother, from a virological and biochemical perspective, and the child. If the child has any lesions on the lips or oral mucosa or if the mother has nipple fissures, breastfeeding should be temporarily interrupted [21,23].

### 4.4. Risk Factors

Although the independent effects of HBV and HCV infections, as well as smoking, on the risk of hepatocellular carcinoma (HCC), have been established, the possible interaction between these factors is not well characterized [24,25]. Regarding the data from the present study, the analysis can indicate an increased risk of hepatocarcinoma for half of the patients and a very high risk for the other half. Some studies have observed an association between smoking and HCC only among HBV-negative individuals, while other studies have reported associations in HBV carriers, and yet other studies have not reported any interaction [25,26]. From a meta-analysis in 2010, no studies were provided regarding the interaction between cigarette smoking and combined HBV and HCV infections [24]. Overall, compared with HBV-negative non-smokers, the risk of HCC was 1.87 for HBV-negative smokers, 15.8 for HBV-positive non-smokers, and 21.6 for HBV-positive smokers. These results suggested more than an additive interaction between these two risk factors, smoking and HBV infection, for HCC [24].

Regarding the risk of horizontal transmission, in our study, most of the responsible surgical interventions are those performed in the dental office (3.4%). To prevent HBV transmission, it is essential to adopt standard precautionary measures, including the use of personal protective equipment in healthcare settings; practicing safe methods for injections, tattoos, and piercings; and avoiding the sharing of personal items that may meet blood. Vaccination against hepatitis B is the most effective method of preventing infection. For three patients, familial transmission was noted, given the positive history of the father and the negative history of the mother for HBV infection. This can occur either using personal items (razors, toothbrushes, scissors, tweezers, sewing needles, glucometers) or through close interpersonal contact (abrasions, household accidents with cuts).

Therefore, it is important to provide a rigorous education to all family members about regular HBV testing to promptly identify and treat any new cases, educate them on hygiene and preventive measures to reduce the risk of transmission, and avoid direct contact with the blood or open wounds of infected persons without appropriate protective measures.

## 5. Conclusions

Vertical transmission continues to be a significant concern, particularly in regions where the disease is widespread. The likelihood of vertical transmission can be greatly minimized through effective preventive strategies. Nonetheless, if antiviral treatment is not administered during pregnancy to lower the viral load, or if the newborn does not receive the first dose of the hepatitis B vaccine and HBIG within 12 h after birth, the risk of transmission remains elevated. Effective prevention of vertical transmission of HBV in children hinges on comprehensive prenatal screening, timely immunoprophylaxis, and, where indicated, antiviral therapy for pregnant women with high viral loads. By adopting these strategies, healthcare providers can significantly reduce the burden of chronic hepatitis B, particularly in areas where the disease is endemic. Ongoing research and improved global health policies remain pivotal in eradicating HBV transmission and improving outcomes in affected populations.

## 6. Limitations

As this is a retrospective study, there are limitations, such as incomplete data sets and potentially inaccurate records, along with geographical limitations. One of the primary limitations of this study is the reliance on historical medical records, which may be incomplete or inconsistently documented. Missing data regarding critical variables, such as maternal viral load, the timing of hepatitis B vaccination, and the exact timing of transmission events (whether vertical or horizontal), could impact the accuracy of the analysis. A significant limitation is the absence of data on maternal HBV DNA levels, a key predictor of vertical transmission risk. Furthermore, the study focuses on a specific region, likely northeastern Romania, and may not be representative of the entire country. The inclusion of both urban and rural populations introduces variability in healthcare access, vaccination coverage, and disease management practices. Although hepatitis B vaccination has been incorporated into the national immunization program, variability in vaccination coverage—such as missed or delayed doses—remains. Without precise vaccination data, it is challenging to assess the protective efficacy of the vaccine and its role in preventing transmission. Future studies should include more specific details, like maternal HBV DNA levels and possibly antiviral treatment during pregnancy, to enhance accuracy and relevance.

## Figures and Tables

**Table 1:** Prevalence of patients by age groups tested for HBV both in the active group and in the control group.

Age Groups	Active	Control	Total
0–1 yrs	30 (20.3%)	222 (43.9%)	252 (38.5%)
2–5 yrs	39 (26.4%)	107 (21.1.%)	146 (22.3%)
6–10 yrs	34 (23.0%)	67 (13.2%)	101 (15.4%)
11–14 yrs	28 (18.9%)	57 (11.3%)	85 (13.0%)
15–18 yrs	17 (11.5%)	53 (10.5%)	70 (10.7%)
Total	148 (100%)	506 (100%)	654 (100%)

**Table 2:** Distribution of patients based on social status (family/foster care/adoption).

Active Group Study	n	%
Adoption	2	1.4
Family	129	87.2
Foster care	17	11.5
Total	148	100

**Table 3:** Distribution of patients regarding the type of birth.

Type of Birth	Active Lot	Control Lot	Total
C—section	29 (19.6%)	138 (27.3%)	167 (25.5%)
Natural	119 (80.4%)	328 (64.3%)	447 (68.3%)
Unknown	0 (0.0%)	40 (7.9%)	40 (6.1%)
Total	148 (100%)	506 (100%)	654 (100%)

**Table 4:** Distribution of patients based on family history.

Family History	Active Lot	Control Lot	Total
Mother	Pearson Chi-square = 437.437, *p* < 0.001		
Absent	27 (18.2%)	407 (80.4%)	434 (66.4%)
HBV	121 (81.8%)	14 (2.8%)	135 (20.6%)
Other	0 (0.0%)	70 (13.8%)	70 (10.7%)
Unknown	0 (0.0%)	15 (3.05%)	15 (2.3%)
Father	Pearson Chi-square = 52.332, *p* < 0.001		
Absent	104 (70.3%)	440 (87.0%)	544 (83.2%)
HBV	21 (14.2%)	6 (1.2%)	27 (4.1%)
Other	15 (10.1%)	43 (8.5%)	58 (8.9%)
Unknown	8 (5.4%)	17 (3.4%)	25 (3.8%)
Grandparents	Pearson Chi-square = 34.720, *p* < 0.001		
Absent	138 (93.2%)	506 (100%)	644 (98.5%)
HBV	10 (6.8%)	0 (0.0%)	10 (1.5%)
Siblings	Pearson Chi-square = 171.417, *p* < 0.001		
No siblings	31 (20.9%)	238 (47.0%)	269 (41.1%)
Siblings with HBV	62 (41.9%)	16 (3.2%)	78 (11.9%)
Healthy siblings	50 (33.8%)	232 (45.8%)	282 (43.1%)
Healthy + HBV-infected siblings	5 (3.4%)	9 (1.8%)	14 (2.1%)
Unknown	0 (0.0%)	11 (2.2%)	11 (1.7%)
Total	148 (100%)	506 (100%)	654 (100%)

**Table 5:** Distribution of patients regarding the vaccination status.

Vaccination Status	Active Lot	Control Lot	Total
Pearson Chi-Square = 17,066/*p* < 0.001 **			
Yes	130 (87.8%)	418 (82.6%)	548 (83.8%)
No	1 (0.7%)	11 (2.2%)	12 (1.8%)
Incomplete	17 (11.5%)	35 (6.9%)	52 (8.0%)
Unknown	0 (0.0%)	42 (8.3%)	42 (6.4%)
Total	148 (100%)	506 (100%)	654 (100%)

** indicates a highly statistically significant difference in the vaccination status distribution between the active and control lots.

**Table 6:** Distribution of patients from HBsAg-positive mothers. Frequency of administration of hepatitis B immunoglobulin in children from HBsAg positive mothers.

HBV Immunoglobulin at Birth	Family History—Mother		
		HBV	
Pearson Chi-Square = 22,159/*p* < 0.001 **			
Yes		5 (4.1%)	
No		32 (26.4%)	
Unknown		84 (69.4%)	
Total		121 (100%)	

** suggests that there is a highly statistically significant difference in the administration of HBV immunoglobulin at birth among the different categories of maternal HBV status.

**Table 7:** Distribution of patients based on smoking/non-smoking environment.

Smoking Environment	Active Lot	Control Lot	Total
Pearson Chi-Square = 25,623/*p* < 0.001 **			
No	74 (50.0%)	265 (55.3%)	339 (54.1%)
Yes	74 (50.0%)	160 (33.4%)	234 (37.3%)
Unknown	0 (0.0%)	54 (11.3%)	54 (8.6%)
Total	148 (100%)	479 (100%)	627 (100%)

** indicates a highly statistically significant difference in the distribution of smoking environments between the active and control lots.

**Table 8:** Distribution of patients regarding risk factors.

Risk Factors	n	%
Adenoidectomy	4	2.7
Tonsillectomy	2	1.4
Dental interventions	5	3.4
Appendectomy	2	1.4
Fractures	2	1.4
Frequent hospitalizations	6	4.1
Frenectomy	1	0.7
Other surgical interventions	3	2.0
Blood transfusion	1	0.7
Smoking environment	74	50.0
Polypectomy	1	0.7
Intrafamilial transmission	3	2.0
Vertical transmission	121	81.7
Horizontal transmission	27	18.2
Total	148	100

**Table 9:** The univariate (Odds Ratio) and multivariate binary logistic regression analyses of risk factors.

Risk Factors	Active Lot (N, %)	Control Lot (N, %)	Total (N, %)	Pearson-Chi Square	Odds Ratio (95% CI)
FR1: HBsAg + mother	121 (81.8)	14 (2.9)	135 (21.1)	Chi2 = 424,907 *p* < 0.001 **	152,690
FR2: Incomplete or no vaccination	18 (12.2)	46 (9.9)	64 (10.5)	Chi2 = 0.606 *p* = 0.436	-
FR3: Natural birth	119 (80.4)	328 (70.4)	447 (72.8)	Chi2 = 5694 *p* = 0.017 *	1726
FR4: Respiratory diseases	75 (50.7)	166 (32.8)	241 (36.9)	Chi2 = 15,712 *p* < 0.001 **	2104
FR5: Endocrine diseases	12 (8.1)	18 (3.6)	30 (4.6)	Chi2 = 5418 *p* = 0.020 *	2392
FR6: Nutrition and metabolic diseases	87 (58.8)	193 (38.1)	280 (42.8)	Chi2 = 19,927 *p* < 0.001 **	2313
FR7: Renal diseases	13 (8.8)	18 (3.6)	31 (4.7)	Chi2 = 6927 *p* = 0.008 **	2611
FR8: Surgical interventions	36 (24.3)	68 (3.4)	104 (15.9)	Chi2 = 10,146 *p* = 0.001 **	2070

* indicates that the results are statistically significant. ** suggests that the results are highly statistically significant.

## Data Availability

The original contributions presented in the study are included in the article; further inquiries can be directed to the corresponding author.
